# The expression of SAH, IL-1β, Hcy, TNF-α and BDNF in coronary heart disease and its relationship with the severity of coronary stenosis

**DOI:** 10.1186/s12872-021-02388-6

**Published:** 2022-03-13

**Authors:** You Wu, Lin Wang, Yu Zhan, Zhixin Zhang, Dan Chen, Yang Xiang, Cui Xie

**Affiliations:** Department of Cardiology, Renmin Hospital, Hubei University of Medicine, No. 39 Chaoyang Middle Road, Shiyan, 442000 Hubei China

**Keywords:** S-adenosylhomocysteine, Serum homocysteine, Inflammatory cytokines, Coronary heart disease, Correlation

## Abstract

**Background:**

To investigate the expression of serum S-adenosylhomocysteine (SAH), interleukin-1β (IL-1β), serum homocysteine (Hcy), tumor necrosis factor-α (TNF-α) and brain derived neurotrophic factor (BDNF) in coronary heart disease and their relationship with the degree of coronary artery disease.

**Methods:**

A total of 132 patients with coronary heart disease (CHD) from March 2020 to April 2021 were included in this retrospective study. The experimental group was composed of CHD patients, including single-vascular group (46 cases), dual-vascular group (49 cases), and multi-vascular group (37 cases). 145 healthy subjects during the same period for physical examination constituted the control group.

**Results:**

The levels of SAH, IL-1β, Hcy, TNF-α and BDNF in single-vascular group, dual-vascular group and multi-vascular group were higher than that in control group, and the differences were statistically significant (*P* < 0.05). The serum levels of SAH, IL-1β, Hcy, TNF-α and BDNF in multi-vascular group were higher than those in single-vascular group and dual-vascular group, and the serum levels of SAH, IL-1β, Hcy, TNF-α and BDNF in dual-vascular group were higher than those in single-vascular group, with statistical significance (*P* < 0.05). Kendall’s tau-b correlation showed that the levels of SAH, IL-1β, Hcy, TNF-α and BDNF were positively correlated with the number of stenosis vessels (r = 0.421, 0.533, 0.301, 0.265, 0.678, *P* = 0.016, 0.009, 0.023, 0.036, 0.004).

**Conclusion:**

SAH, IL-1β, Hcy, TNF-α and BDNF in serum of patients with CHD can be used as effective biological indicators to monitor the degree of CHD and severity of coronary stenosis.

## Background

Coronary heart disease (CHD) is a common clinical heart disease. It refers to vascular stenosis or obstruction caused by atherosclerotic lesions in the coronary arteries, leading to myocardial dysfunction and/or organic changes caused by myocardial ischemia, hypoxia or necrosis. It has high morbidity, high mortality, high recurrence rate and high mortality, which has a serious impact on the daily life and life of patients [1]. At present, there are many methods for clinical diagnosis of coronary heart disease, but these methods are expensive, traumatic, and have certain limitations [2]. Hence, the use of serological indicators to detect and evaluate the condition of coronary heart disease still has important clinical value for diagnosis and condition monitoring.

The occurrence of coronary heart disease is closely related to many factors. S-adenosylhomocysteine (SAH) and serum homocysteine (Hcy) are both metabolites of methionine, which are conducive to the generation of oxygen free radicals to cause vascular endothelial cell damage, and are multifunctional injury factors and chronic inflammatory factors involved in the process of coronary atherosclerosis [3]. Interleukin-1β (IL-1β) promotes the formation of coronary atherosclerosis [4]. Tumor necrosis factor -α (TNF-α) is mainly produced by monocytes and macrophages, and plays an important role in the formation of atherosclerosis [5]. Brain-derived neurotrophic factor (BDNF) is a pro-angiogenic factor, which can induce angiogenesis in myocardial ischemic tissue and induce inflammation [6].

Studies have confirmed that the changes in the levels of SAH, IL-1β, Hcy, TNF-α, and BDNF in the human body are closely related to cardiovascular diseases [5–9]. However, there are few studies on the expression of serum SAH, IL-1β, Hcy, TNF-α, and BDNF in coronary heart disease and their relationship with the extent of coronary artery stenosis. Based on this, this study aimed to explore the expression of serum SAH, IL-1β, Hcy, TNF-α, BDNF in coronary heart disease and the relationship with the degree of coronary artery disease.

## Methods

### Subjects

This study included 132 patients with coronary heart disease and 145 healthy subjects who were admitted to our hospital from March 2020 to April 2021. Patients with coronary heart disease were set as the study group, and healthy patients were set as the control group. According to the number of coronary artery stenosis indicated by coronary angiography, they were divided into single-vessel group (46 cases), double-vessel group (49 cases) and three-vessel group (37 cases).

Inclusion criteria: (1) patients that meet the diagnostic criteria of coronary heart disease in "Diagnostic Criteria for Coronary Atherosclerotic Heart Disease" [10], and coronary angiography shows that the inner diameter of any one of the left main trunk, left circumflex artery, left anterior descending artery, and right coronary artery stenosis degree ≥ 50% or left main stem stenosis > 30%; (2) no immune system diseases; (3) no serious disease of other organs; (4) healthy mental state, and good compliance. Exclusion criteria: (1) severe coagulopathy; (2) acute myocardial infarction; (3) hypothyroidism and hyperthyroidism; (4) congenital heart disease; (5) liver and kidney failure or cerebrovascular disease; (6) receiving percutaneous coronary intervention or coronary artery bypass grafting; (7) recent use of drugs such as glucocorticoids or growth hormones; (8) allergic to iodine or contrast agents; (9) acute pulmonary edema. This study complies with the relevant requirements of the "Declaration of Helsinki of the World Medical Association". The study was approved by the Ethics Committee of the XXXX Hospital, Hubei University (No. HBPH-20-02-07). Patients provided written informed consent.

### Biochemical measurements

Sample collection and processing: 5–8 ml of fasting cubital venous blood samples were collected from the two groups in the morning, centrifuged at 3000r/min for 10 min at 4 °C, the serum samples were then separated and stored in an ultra-low temperature refrigerator at − 80 °C for testing. Total cholesterol (TC), high-density lipoprotein cholesterol (HDL-C), low-density lipoprotein cholesterol (LDL-C) were measured by AU 5800 automatic biochemical analyzer. Creatine kinase isoenzyme (CK-MB) was detected by electrochemiluminescence method (Shanghai Tianneng Technology Co., Ltd), IL-1β, Hcy, TNF-α, BDNF in plasma samples were tested by enzyme-linked immunosorbent assay (ELISA) (Shanghai Enzyme Link Biotechnology Co., Ltd), SAH was detected by enzyme continuous cycle colorimetric method (GenMed Inc).

### Statistical analysis

Statistical analysis was performed using SPSS 23.0 software. Measurement data were expressed as mean ± SD, and t test was used for measurement data between each group, counting data were compared by chi-square test. All data were tested for normality and homogeneity of variance. The serum levels of SAH, IL-1β, Hcy, TNF-α, and BDNF in each group were compared, and the correlation between SAH, IL-1β, Hcy, TNF-α, BDNF levels and the number of coronary heart disease lesions was determined by Kendall’s tau-b correlation. The difference was statistically significant with *P* < 0.05.

## Results

### General data

There was no significant difference in age and gender of each group (*P* > 0.05). Body mass index (BMI), smoking history, complication of hypertension, complication of diabetes, TC and LDL-C levels of single-vascular group, dual-vascular group and multi-vascular group were higher than those in the control group, meanwhile, the levels of HDL-C were lower than those in the control group, and the differences were statistically significant (*P* < 0.05). There were no significant differences in BMI, smoking history, course of disease, complication of hypertension, complication of diabetes, TC, LDL-C and HDL-C levels in single-vascular group, dual-vascular group and multi-vascular group (*P* > 0.05) (Table 1).

**Table 1:** General information of each group

Item	Single-vascular group (n = 46)	Dual-vascular group (n = 49)	Multi-vascular group (n = 37)	Control group (n = 145)
Age (years)	48.98 ± 9.83	51.02 ± 10.21	53.29 ± 10.32	51.71 ± 10.33
Gender (male/female)	21/25	20/29	15/22	62/83
Course of disease (years)	4.23 ± 1.22	4.33 ± 1.29	4.47 ± 1.35	–
BMI (kg/m^2^)	24.57 ± 2.39*	24.97 ± 2.59*	25.65 ± 2.78*	22.19 ± 2.29
Complication of hypertension (n)	20*	22*	18*	0
Smoking history (n)	7*	7*	6*	2
Complication of diabetes (n)	15*	17*	13*	0
TC (mmol/L)	4.64 ± 1.24*	4.89 ± 1.32*	4.97 ± 1.38*	3.12 ± 1.01
HDL-C (mmol/L)	1.12 ± 0.31*	1.08 ± 0.35*	1.02 ± 0.36*	1.48 ± 0.29
LDL-C (mmol/L)	2.48 ± 0.63*	2.56 ± 0.61*	2.59 ± 0.63*	1.87 ± 0.61

*BMI* body mass index, *TC* total cholesterol, *HDL-C* high density lipoprotein cholesterol, *LDL-C* low density lipoprotein cholesterol

*Compared with control group, *P* < 0.05

### Comparison of serum SAH, IL-1β, Hcy, TNF-α, BDNF levels in each group

The levels of SAH, IL-1β, Hcy, TNF-α and BDNF in single-vascular group, dual-vascular group and multi-vascular group were higher than those in the control group, and the difference was statistically significant (*P* < 0.05). The levels of SAH, IL-1β, Hcy, TNF-α and BDNF in the multi-vascular group were higher than those in the single-vascular group and dual-vascular group (*P* < 0.05).The levels of serum SAH, IL-1β, Hcy, TNF-α and BDNF in dual-vascular group were higher than that in single-vessel group, and the difference was statistically significant (*P* < 0.05) (Table 2).

**Table 2:** Comparison of serum SAH, IL-1β, Hcy, TNF-α, BDNF levels

Item	Single-vascular group (n = 46)	Dual-vascular group (n = 49)	Multi-vascular group (n = 37)	Control group (n = 145)
SAH (nmol/L)	28.78 ± 3.76*	38.94 ± 4.11*^#^	49.84 ± 4.02*^#@^	20.01 ± 3.21
IL-1β (ng/L)	31.23 ± 4.18*	34.39 ± 4.29*^#^	37.93 ± 4.39*^#@^	15.83 ± 3.46
Hcy (μmol/L)	23.39 ± 4.02*	29.89 ± 4.11*^#^	36.84 ± 4.18*^#@^	17.02 ± 3.98
TNF-α (pg/mL)	13.29 ± 3.01*	16.73 ± 3.18*^#^	21.29 ± 3.29*^#@^	3.49 ± 1.02
BDNF (ng/mL)	893.39 ± 67.38*	955.94 ± 66.39*^#^	999.93 ± 69.38*^#@^	801.28 ± 62.19

*SAH* S-adenosylhomocysteine, *IL-1β* interleukin-1β, *Hcy* homocysteine, *TNF-α* tumor necrosis factor-α, *BDNF* brain derived neurotrophic factor

*Compared with control group, *P* < 0.05

^#^Compared with single-vascular group, *P* < 0.05

^@^Compared with dual-vascular group, *P* < 0.05

### Correlation analysis of SAH, IL-1β, Hcy, TNF-α, BDNF levels and the number of stenosis vessels in coronary heart disease

The correlation analysis of SAH, IL-1β, Hcy, TNF-α, BDNF and the number of coronary artery stenosis was conducted by Kendall’s tau-b correlation, respectively. It was suggested that SAH, IL-1β, Hcy, TNF-α, BDNF were positively correlated with the number of coronary artery stenosis vessels(r = 0.421, 0.533, 0.301, 0.265, 0.678, *P* = 0.016、0.009、0.023、0.036、0.004).

## Discussion

Studies indicated that the number of patients who die from CHD is as high as 7 million each year [11]. With the improvement of people's living standard and the aggravation of population aging process, the incidence and mortality of CHD are increasing year by year, and the trend is younger, which has become a major cardiovascular disease harmful to health [12]. Fatigue, overeating, strenuous exercise, acute circulatory failure, and excessive mood swings are the main causes of CHD. The main clinical manifestations are dizziness, chest tightness, dyspnea, belching and other symptoms; pathological manifestations are mainly hypoxia and ischemia in the heart of the patient caused by clogged blood vessels. Since CHD causes greater physical and psychological damage to patients, active prevention has important clinical significance. The pathogenesis of coronary heart disease is the occurrence of atherosclerosis in the coronary arteries, which leads to stenosis or occlusion of the official cavity, and causes myocardial ischemia, hypoxia or necrosis. Diabetes mellitus, hypertension, dyslipidemia, smoking and other factors are closely related to atherosclerosis [13]. This study used a single factor to analyze the relationship between the above factors and coronary heart disease. The results showed that diabetes, hypertension, dyslipidemia, smoking and other factors in the CHD group were significantly higher than those in the healthy group, consistent with previous research results, indicating that BMI, smoking history, history of hypertension, history of diabetes mellitus, TC and LDL-C levels are closely related to coronary heart disease.

In order to predict the severity of coronary heart disease, serological indicators have been widely studied as a non-invasive examination. A cross-sectional study conducted by Huang et al. confirmed that higher levels of SAH are associated with a higher risk of cardiovascular events and can be used as a risk predictor for diagnosing cardiovascular disease [14]. Xiao et al. [15] found that SAH level can be used as an effective predictor of cardiovascular risk. IL-1β is a form of interleukin-1 (IL-1) in the body, which can eventually activate the Rel family transcription factor nuclear factor kB (NF-kB) and is mainly involved in the regulation of physiological processes such as immunity, inflammatory response and cell differentiation. Recent studies have confirmed that the secretion of IL-1β increases the risk of cardiovascular diseases, with great significance for the clinical diagnosis and condition assessment and monitoring of coronary heart disease [16]. Hcy is a thiol groups of amino acid, methionine metabolism is intermediate, methylation and antioxidant levels associated with the human body, is a new and important risk factor of atherosclerosis and its toxic to vascular endothelial function, and can increase plaque ulnerability, thrombosis, to increase the risk of coronary heart disease occurs [17]. Dong et al. [17] found that the serum Hcy level increased significantly with the exacerbation of CHD. Ni et al. [18] found the serum Hcy level of patients with CHD was positively correlated with the degree of coronary artery stenosis. TNF-α promotes inflammation by stimulating the production of inflammatory cytokines, and mediates inflammation and regulates immune function. It is mainly produced by monocytes and macrophages, and can play a role in damaging vascular endothelial cells, forming thrombus, causing vascular blockage, inducing apoptosis of myocardial cells, and finally leading to physiological dysfunction of myocardial muscle. The expression of TNF-α is not obvious or not expressed in normal myocardial tissue. Myocardial infarction can lead to the generation of TNF-α in myocardium, and TNF-α can induce cytotoxic effect in cells, and induce cardiac insufficiency and ventricular remodeling. Yuan et al. [19] indicated that the expression level of TNF-α in elderly patients with coronary heart disease is high, which is of great clinical significance for clinical diagnosis, prognosis and prevention. Li et al. [20] also found that the combined detection of serum IL-6, TNF-α and monocyte chemoattractant protein-1 (MCP-1) can increase the positive detection rate of CHD, which is of great significance for the early diagnosis of CHD patients.

BDNF is a protein synthesized in the brain and is widely found in the central and peripheral nervous systems. In the development of the nervous system, BDNF plays an important role in the survival, growth and development of neurons. At the same time, it has biological effects such as improving the pathological state of neurons, preventing the injury and death of neurons, and promoting the regeneration and differentiation of damaged neurons. Tschorn M et al. [21] found that BDNF was a new pro-angiogenic factor, which can inhibit myocardial cell apoptosis, promote endothelial cell proliferation and migration in ischemic sites, and induce angiogenesis in ischemic tissues, and BDNF was highly expressed in atherosclerosis and ischemic heart disease. Liu et al. [22] also confirmed that SAH level was a relatively sensitive biomarker for atherosclerosis. Luo et al. [23] found that S-adenosine homocysteine hydrolase (SAHH) inhibitors and short hairpin RNA (shRNA) interference induced the increase of SAH levels, and induced the proliferation and migration of smooth muscle cells through oxidative stress-extracellular signal-regulated protein kinase 1 and 2 pathways. Oxidative stress can explain the mechanism of SAH leading to atherosclerosis. Recent studies [24] indicated that SAH promotes atherosclerosis and is associated with epigenetic regulation of ER stress. The results of this study indicated that serum levels of SAH, IL-1β, Hcy, TNF-α and BDNF were highly expressed in patients with CHD, and were positively correlated with the number of vessels with stenosis. Based on the findings of the present study, the levels of SAH, IL-1β, Hcy, TNF-α and BDNF can predict the severity of coronary heart disease.

There were also some limitations in this study. First, the sample size of this study was small, and there was a lack of multi-center randomized controlled trial. Second, this study lacked follow-up results of serological indicators in patients after treatment. Further studies are needed to investigate the association between inflammatory indicators and the prognosis of CHD.

## Conclusion

In conclusion, the serum levels of SAH, IL-1β, Hcy, TNF-α and BDNF were increased in patients with CHD, and the levels of SAH, IL-1β, Hcy, TNF-α and BDNF were positively correlated with the number of stenosis vessels, which could be used as biochemical indicators for monitoring the condition of CHD.

## Data Availability

The datasets used and/or analyzed during the current study are available from the corresponding author on reasonable request.

## Abbreviations

BDNF: Brain derived neurotrophic factor
CHD: Coronary heart disease
Hcy: Homocysteine
IL-1β: Interleukin-1β
SAH: S-adenosylhomocysteine
TNF-α: Tumor necrosis factor-α
